# Long-Term Outcomes in NSTEMI Patients Based on Coronary TIMI Flow State on Presentation

**DOI:** 10.3390/jcm15124486

**Published:** 2026-06-10

**Authors:** Tarek Abdeldayem, Hilal Khan, Mohamed Farag, Ioakim Spyridopoulos, Mohammad Alkhalil, Scott Wilkes, Emmanouil S. Brilakis, Bilal Bawamia, Mohaned Egred

**Affiliations:** 1 Cardiothoracic Centre, Freeman Hospital, Newcastle upon Tyne NE7 7DN, UK; tarek.abdeldayem1@nhs.net (T.A.); mohammad.alkhalil@nhs.net (M.A.);; 2 Translational and Clinical Research Institute, Newcastle University, Newcastle upon Tyne NE7 7DN, UK; 3 School of Medicine, University of Sunderland, Sunderland SR1 3SD, UK; 4 Minneapolis Heart Institute, Minneapolis, MN 55407, USA

**Keywords:** coronary artery disease, non-ST elevation myocardial infarction, TIMI flow, angioplasty, PCI, mortality

## Abstract

**Background/Objectives**: People with non-ST-segment elevation myocardial infarction (NSTEMI) with an occluded culprit vessel represent a unique subset of patients; however, their long-term outcomes remain unclear. This study aimed to compare 5-year mortality between NSTEMI patients treated with percutaneous coronary intervention (PCI) based on TIMI flow states in the culprit vessel on presentation (TIMI 0-1 compared to TIMI 2-3). **Methods**: A retrospective analysis of prospectively collected data of all NSTEMI patients who underwent PCI from 2012 to 2019 at a tertiary cardiac center (The Freeman Hospital, Newcastle-Upon-Tyne, UK) with follow up for 5 years until January 2024. Patients were identified from the database and categorized based on pre-procedural TIMI flow in the culprit vessel. A propensity score was used to pair TIMI 0-1 patients with a matched cohort of TIMI 2-3 patients. The primary outcome was 5-year all-cause mortality. **Results**: A total of 775 patients with TIMI 0-1 flow were matched with 750 patients who had TIMI 2-3 flow. Patients with TIMI 0-1 flow were more likely to have transient ST elevation (24% vs. 18%, *p* < 0.001) or Q waves (4% vs. 1%, *p* < 0.001) compared with patients who had TIMI 2-3 flow. They were also more likely to have moderately to severely impaired left ventricular systolic function compared with patients with TIMI 2-3 flow (21% vs. 16%, *p* = 0.01). In-hospital mortality (1.2% vs. 1.2%, *p* = NS), 1-year mortality (5% vs. 6.9%, *p* = NS), and 5-year mortality (16% vs. 18%, *p* = 0.34) were not significantly different between the two groups. The use of glycoprotein IIb/IIIa antagonists was associated with lower mortality, HR 0.64 (0.46 to 0.87). **Conclusions**: NSTEMI patients with occluded culprit vessels who underwent PCI had similar in-hospital and long-term outcomes to patients with patent culprit vessels. The use of glycoprotein IIb/IIIa inhibitors appears to be associated with lower mortality.

## 1. Introduction

Non-ST-segment elevation myocardial infarction (NSTEMI) is the most prevalent presentation of acute coronary syndromes (ACSs) [1]. Unlike ST-segment elevation myocardial infarction (STEMI), most NSTEMI patients present with a patent culprit vessel which is defined as Thrombolysis in Myocardial Infarction (TIMI) 2 to 3 flow. However, a subset of NSTEMI patients present with complete occlusion in the culprit vessel, defined as TIMI grade 0 to 1 flow [1,2].

Patients with NSTEMI who have an occluded culprit vessel in a major epicardial coronary artery and do not have ST elevation on their ECG often undergo delayed percutaneous coronary intervention (PCI) [3,4]. There are conflicting data on the incidence of short-term major adverse cardiovascular events (MACEs), with some studies demonstrating no difference and others indicating higher MACE rates in patients with occluded culprit vessels [5,6]. Previous meta-analyses have shown higher mortality and risk of recurrent myocardial infarction (MI) in this patient cohort; however, the impact of PCI therapy was not assessed and there is limited long-term data regarding PCI outcomes in these patients [7,8].

We compared the immediate and long-term outcomes of NSTEMI patients treated with PCI who presented with occluded vs. patent culprit vessels.

## 2. Methods

### 2.1. Study Design and Population

This is a retrospective analysis of prospectively collected data on all patients undergoing coronary intervention at a large tertiary center. Data for all NSTEMI patients admitted to the Freeman Hospital from 2012 to 2019 were acquired from the national institute for cardiovascular research dataset (NICOR) and combined with local mortality data for each patient. Data were collected as part of a national audit.

Ethical review and approval were waived for this study as this is a retrospective study. The data are anonymous and available to the public. Informed Consent was also waived due to the anonymous data in this retrospective study.

### 2.2. Baseline and Procedural Characteristics

Patient and procedural characteristics were extracted from the database. Myocardial infarction (MI) was defined as per the fourth universal definition of myocardial infarction. TIMI flow status was defined upon review of patient angiograms. Two experienced cardiologists reviewed the angiograms and where there was disagreement, a senior cardiologist adjudicated. Mortality data were extracted from local hospital records and linked with cases from the database.

### 2.3. Study Groups

The TIMI flow status observed in the culprit vessel prior to intervention served as the basis for categorizing patients into two distinct groups: those demonstrating TIMI 2-3 flow (patent culprit vessels), and those exhibiting TIMI 0-1 flow (occluded culprit vessels, as defined previously).

### 2.4. Outcomes

The primary outcome of this study was 5-year all-cause mortality. Secondary outcomes included death, recurrent MI, stroke, arterial complications, cardiac tamponade, and early mortality.

### 2.5. Statistical Analysis

IBM SPSS (Version 27) was used for statistical analysis. Propensity matching was performed in SPSS using baseline characteristics which formed traditional prognostic risk factors for coronary artery disease such as age, gender, history of PCI, history of MI, history of CABG, history of peripheral artery disease, history of stroke, history of chronic kidney disease, history of hypertension, history of hypercholesterolemia, family history of coronary artery disease, history of diabetes and current smoker, and procedural characteristics such as presence of cardiogenic shock, multivessel disease and need for complex calcium modification. Matching was performed without replacement and with the caliper set at 0.1 using the fuzzy extension in SPSS.

Continuous variables were expressed as mean ± standard deviation or median (interquartile range) and compared using Student’s *t*-test or Wilcoxon’s rank-sum test, as appropriate. Categorical variables were expressed as percentages and compared using the chi square test. Kaplan–Meier survival analysis was used to estimate all-cause mortality in both groups and the log rank test was used to compare the event rates between the two groups. Cox regression analysis was performed on all key risk factors in the baseline and procedural characteristics. A sensitivity analysis was also performed between TIMI 0 and TIMI 1-3 in order to test the robustness of the comparison.

## 3. Results

**Figure 1:**
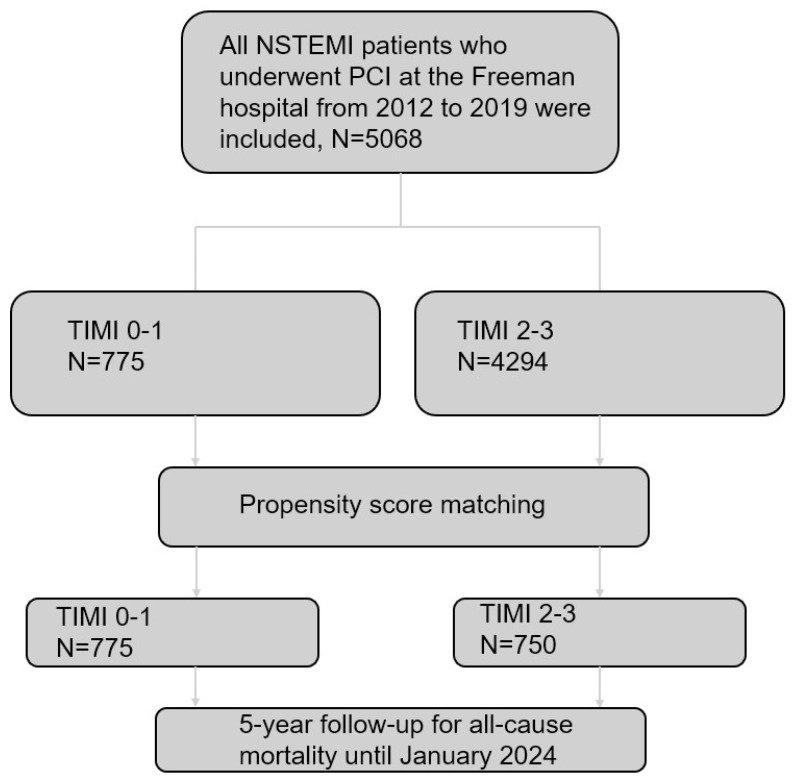
Flowchart depicting patient inclusion in the study.

A total of 5068 patients treated with PCI were included, of whom 775 patients exhibited TIMI 0-1 flow and 4293 demonstrated TIMI 2-3 flow. After propensity matching 750 patients in the TIMI 2-3 group were included in the final analysis (Figure 1).

### 3.1. Baseline Characteristics

Baseline characteristics are presented in (Table 1).

The median age in the TIMI 0-1 group was 63 (21-96) years vs. 65 (30-95) years in the TIMI 2-3 group. The groups were well matched except for slightly more strokes in the TIMI 2-3 group (8% vs. 5%, *p*= 0.03). Patients with TIMI 2-3 flow were more likely to have no ECG changes compared to TIMI 0-1 flow (31% vs. 21%, *p* < 0.001). Patients with TIMI 0-1 flow were more likely to have transient ST elevation (24% vs. 18%, *p* < 0.001) and Q waves on ECG (4% vs. 1%, *p* < 0.001) compared with TIMI 2-3 flow patients. Patients with TIMI 0-1 were also more likely to have moderately–severely impaired left ventricular systolic function (21% vs. 16%, *p* = 0.01).

### 3.2. Procedural Characteristics

The procedural characteristics in each group are shown in Table 2.

There were more patients presenting with left main as the culprit vessel in the TIMI 2-3 group compared with the TIMI 0-1 group (7% vs. 1%, *p* = 0.04). There were more patients with RCA culprit involvement in the TIMI 0-1 group compared with the TIMI 2-3 group (27% vs. 22%, *p* = 0.03). Coronary physiology was more likely to be performed in the TIMI 2-3 group compared with the TIMI 0-1 group (10% vs. 3%, *p* < 0.001). There was greater use of thrombectomy devices in TIMI 0-1 compared to TIMI 2-3 (13% vs. 8%, *p* = 0.006) and glycoprotein IIb/IIIa antagonists (36% vs. 28%, *p* < 0.001). TIMI flow in the culprit vessel post PCI was more likely to be TIMI 0 in the TIMI 0-1 compared with the TIMI 2-3 group (8% vs. 0.8%, *p* < 0.001) and more likely to be TIMI 3 in TIMI 2-3 compared with the TIMI 0-1 group (86% vs. 74%, *p* < 0.001).

### 3.3. Outcomes

The in-hospital and longer-term outcomes were not statistically significantly different between the two groups (Table 3).

**Figure 2:**
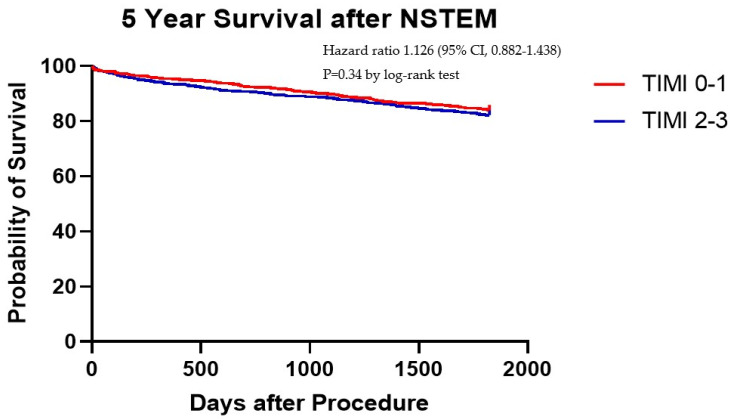
Kaplan–Meier graph of 5-year survival after NSTEMI with TIMI 0-1 shown in red and TIMI 2-3 shown in blue.

The incidence of cerebrovascular events, recurrent myocardial infarction, tamponade, and arterial complications did not differ significantly between the two groups. The Kaplan–Meier survival analysis revealed no statistically significant differences in mortality between the two groups at 1, 6, 12 months and 5 years as shown in Table 3 and Figure 2.

Cox regression survival analysis (Table 4) demonstrated that TIMI 0 flow was not independently associated with higher mortality (HR 1.08, 0.49 to 2.32). However, increasing age, peripheral arterial disease, diabetes, chronic kidney disease, severe left ventricular systolic dysfunction, cardiogenic shock, left main as culprit, left anterior descending artery and right coronary artery as culprit vessels were independently associated with higher mortality over 5 years.

**Figure 3:**
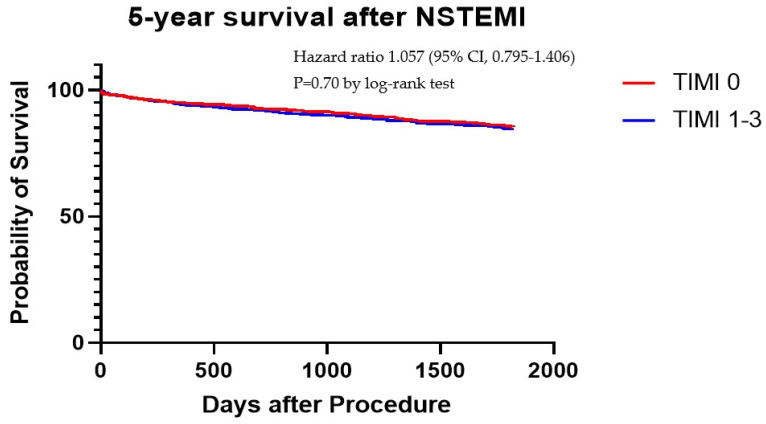
Kaplan–Meier graph of 5-year survival after NSTEMI with TIMI 0 shown in red and TIMI 1-3 shown in blue after repeat propensity matching.

The use of intra-procedure glycoprotein IIb/IIIa antagonists was associated with lower mortality (HR 0.64, 0.46 to 0.87). Sensitivity analysis did not show any difference in outcomes between TIMI 0 vs. TIMI 1-3 after PCI (Figure 3).

## 4. Discussion

The main finding of our study is that NSTEMI patients who presented with an occluded culprit vessel and underwent PCI, had a similar benefit from PCI as patients who presented with TIMI 2-3 flow. This allows us to argue that such patients should undergo revascularization rather than medical treatment.

In the last two decades there has been a substantial reduction in mortality among patients with NSTEMI, due to improvements in medical therapy and timely availability of revascularization [9]. There are subgroups of patients with NSTEMI among whom the long-term outcomes are less clear, such as those who present with occluded culprit vessels (TIMI 0-1 flow). This study provides valuable insights into the long-term outcomes of NSTEMI patients, with occluded culprit vessels treated with PCI.

The two groups in our study were well matched for age, gender and comorbidities. NSTEMI patients who had TIMI 2-3 flow were more likely to have no ECG changes compared with patients with TIMI 0-1 flow who were more likely to have transient ST changes or Q waves on ECG. This is in keeping with the literature that shows many NSTEMI patients presenting with non-diagnostic or normal ECG [10]. However, patients with NSTEMI who have an occluded culprit vessel are more likely to have transient ST changes or pathological Q waves compared to those with patent culprit vessels [11].

NSTEMI patients with TIMI 0-1 flow may represent a subgroup that experiences gradual progression of atheromatous disease in the culprit vessel, leading to acute coronary occlusion [12,13]. This may allow time for ischemic conditioning and collateral circulations to develop which has previously been described in these patients, this may prevent transmural infarction, and lead to atypical ECG presentations that do not meet STEMI criteria [14,15].

Patients with occluded culprit vessels were more likely to present with moderately–severely impaired left ventricular systolic function. These patients have higher troponin rises, in keeping with greater myocardial injury [16]. This does emphasize the importance of earlier identification and treatment in these patients, which may be brought about by a paradigm shift towards an occlusive and non-occlusive myocardial infarction classification over that of the traditional NSTEMI and STEMI criteria [17]. This is also re-enforced by data demonstrating similar longer-term outcomes in patients presenting with classical NSTEMI and STEMI patients [18]. Advances in machine learning models to assist ECG diagnosis has been shown in one study to play a possible role in facilitating early diagnosis and may be one way in which this shift to an occlusive and non-occlusive MI paradigm may be facilitated [19].

There were more patients with left main as the culprit vessel in the TIMI 2-3 group compared to the TIMI 0-1 group and more patients with the right coronary artery involvement in the TIMI 0-1 compared to the TIMI 2-3 group. This is likely explained by the fact that patients with occluded left main are unlikely to survive to hospital admission particularly in the absence of significant collateralization [20]. Overall, the circumflex was most likely to be the culprit vessel in almost 40% of our cases which is in keeping with the literature describing this vessel as the most implicated in occlusive myocardial infarction without ST elevation given the electrocardiographically silent nature of the posterior territory [21,22].

There was more use of coronary physiology in patients with patent culprit vessels, likely due to the ambiguity which can exist in NSTEMI patients with multivessel disease, necessitating coronary physiology to guide revascularization strategies [23]. There was greater use of thrombectomy devices and adjunctive glycoprotein IIb/IIIa antagonists in patients with TIMI 0-1 flow due to the known higher thrombus burden in occluded culprit vessels compared with patent vessels [24].

Patients who present with TIMI 0-1 flow were more likely to have TIMI 0 flow after coronary intervention compared with patients who had TIMI 2-3 flow. Presentation with TIMI 0-1 flow in NSTEMI is associated with a higher risk of TIMI 0 flow post PCI that is explained by a higher thrombus burden and subsequent risk of no reflow and distal embolization [25].

Consistent with prior studies our study revealed no significant differences in in-hospital complications or long-term mortality between the TIMI 0-1 and TIMI 2-3 flow groups treated with PCI [26]. TIMI 0 flow in NSTEMI patients has been associated with comparatively smaller infarct sizes than patients with STEMI and TIMI 0 flow [27]. It is also known that many of these patients present with more gradual disease progression allowing development of more robust collateralization which prevents larger infarcts compared with patients who presented with a STEMI [27]. Other studies have similarly demonstrated no longer-term difference in mortality at follow-up [27,28].

Mortality in our study was largely driven by traditional prognostic risk factors such as older age, peripheral arterial disease, diabetes, chronic kidney disease, cardiogenic shock and severe left ventricular systolic dysfunction [29] as well as culprit vessels involved such as left main, left anterior descending artery and right coronary artery territories having a prognostically relevant effect on mortality whereas the circumflex artery did not.

The left main stem and left anterior descending arteries had the greatest effect on mortality which is unsurprising with the left main stem supplying over 60% of the myocardium and the left anterior descending supplying over 40% from pathological studies [27,30]. Some studies report a greater effect of the circumflex on mortality compared to the right coronary artery and others describe no impact on mortality likely related to the prevalence of dominance in these vessels [30,31].

Glycoprotein IIb/IIIa antagonist administration was associated with lower long-term mortality whereas the use of thrombectomy catheters did not have such effect. This may be due to the ability of pharmacotherapy to act on micro-emboli unlike thrombectomy catheters and is supported by a meta-analysis on glycoprotein IIb/IIIa antagonists that showed associated lower long-term mortality [32]. Early data on thrombus aspiration devices showed improved 30-day outcomes but failed to show durable improvement in outcomes [33,34,35].

Our data shows that unlike in the STEMI setting there is no long-term hazard to reperfusion of the occluded culprit vessel even at a median of 3 days from event to intervention, with identical long-term outcomes to coronary intervention in patients with patent culprit vessels [36]. This contrasts with other registry data that reported higher long-term mortality; however, in these registries, reperfusion was only performed in approximately 80% of patients [8] and a lack of reperfusion therapy may have driven outcomes in these studies, whereas all patients in our study underwent revascularization.

## 5. Limitations

This is a single center, retrospective study with all recognized inherent limitations. Propensity matching was performed to reduce variability; however, residual confounding remains possible. While the TIMI flow assessment can be subjective, there is usually agreement on TIMI 0-1 grade flow among assessors. There have been major changes in the PCI armamentarium and guidelines that occurred during the study period, and it is not clear if these changes would have had any impact on the outcome.

## 6. Conclusions

This study demonstrates no difference in long-term mortality in TIMI 0-1 flow compared with TIMI 2-3 flow in NSTEMI patients treated with PCI. Administration of glycoprotein IIb/IIIa antagonists was associated with lower long-term mortality.

## Figures and Tables

**Table 1:** Baseline characteristics in NSTEMI patients with unmatched TIMI 2-3 flow compared to TIMI 0-1 flow and TIMI 0-1 compared to TIMI 2-3 flow after propensity matching.

	TIMI 2-3 N = 4294	TIMI 0-1 N = 775	*p* Value	TIMI 2-3 N = 750	*p* Value
**Age**	67 (58–76)	63 (55–73)	0.001	65 (56–74)	0.051
**Gender**					
*Male*	2998 (69.8)	591 (76)	<0.001	541 (72)	0.066
*Female*	1296 (30.2)	184 (24)	<0.001	209 (28)	0.066
**Comorbidities**					
*Previous MI*	1320 (30.9)	199 (26)	0.004	219 (29)	0.122
*Previous PCI*	1123 (26.3)	177 (22)	0.010	145 (19)	0.214
*Previous CABG*	239 (5.6)	42 (5)	0.847	52 (7)	0.224
*Diabetes Mellitus*	1121 (26.1)	176 (23)	0.046	168 (22)	0.885
*Chronic Kidney Disease*	165 (3.8)	26 (3)	0.512	33 (4)	0.290
*Hyperlipidemia*	2335 (54.4)	384 (50)	0.013	410 (55)	0.051
*Peripheral Vascular Disease*	353 (8.2)	51 (7)	0.121	67 (9)	0.086
*Hypertension*	2850 (66.4)	453 (59)	<0.001	453 (60)	0.439
*Cerebrovascular Accident*	345 (8.0)	42 (5)	0.012	62 (8)	0.03
*Smoking*	2833 (66.2)	516 (67)	0.932	505 (68)	0.752
**Family History of Ischaemic Heart Disease**	2012 (47.0)	357 (46)	0.462	342 (46)	0.913
**Presenting ECG Changes**					
*No Changes*	1732 (41.1)	159 (21)	<0.001	231 (31)	<0.001
*Transient ST Elevation*	338 (8.0)	184 (24)	<0.001	132 (18)	<0.001
*ST Depression*	656 (15.6)	139 (18)	<0.001	128 (17)	0.701
*T Wave Inversion*	1361 (32.3)	257 (34)	0.520	240 (33)	0.697
*Q Waves*	129 (3.1)	29 (4)	0.297	7 (1)	<0.001
**Left Ventricular Function**					
*Good LVEF > 50*	1021 (23.8)	150 (19)	<0.001	160 (21)	0.337
*Moderate (LVEF 30-50)*	497 (11.6)	160 (21)	<0.001	117 (16)	0.011
*Poor LVEF < 30)*	220 (5.1)	43 (5)	0.623	39 (5)	0.763
*Not Measured*	2556 (59.5)	422 (55)	<0.001	434 (58)	0.179
**Admission Route**					
*Direct admission*	115 (2.7)	35 (5)	0.014	24 (3)	0.168
*Inter-hospital transfer*	3987 (95.0)	718 (94)	0.014	701 (94)	0.832
*Already in cardiac center*	93 (2.2)	12 (2)	0.252	24 (3)	0.055
**Cardiogenic Shock**	39 (0.9)	15 (2)	0.011	16 (2)	0.787
**Time from MI to PCI**	4 (2–6)	3 (1–5)	0.001	4 (3–7)	0.01

Values are median (IQR), n (%), or mean ± SD. TIMI = Thrombolysis in Myocardial Infarction; MI = myocardial infarction; PCI = percutaneous coronary intervention; CABG = Coronary Artery Bypass Grafting; ECG = Electrocardiogram; LVEF = Left Ventricular Ejection Fraction; NS = Not Significant.

**Table 2:** Procedural characteristics in NSTEMI patients with unmatched TIMI 2-3 flow compared to TIMI 0-1 flow and TIMI 0-1 compared to TIMI 2-3 flow after propensity matching.

	TIMI 2-3 N = 4294	TIMI 0-1 N = 775	*p* Value	TIMI 2-3 N = 750	*p* Value
**Arterial Access**					
*Femoral*	536 (12.5)	111 (14)	0.158	122 (16)	0.291
*Radial*	3739 (87.1)	661 (85)	0.245	624 (83)	0.262
**Culprit Artery**					
*Left Main Stem Artery*	402 (10.0)	33 (4.3)	<0.001	50 (7)	0.042
*Left Anterior Descending Artery*	2214 (55.2)	345 (45.3)	<0.001	314 (42.0)	0.452
*Circumflex Artery*	1285 (32.1)	263 (34.5)	0.185	251 (33.5)	0.171
*Right Coronary Artery*	1461 (36.5)	134 (17.3)	0.004	135 (18.0)	0.347
**Mean Vessels Attempted**	1.36 ± 0.72	1.32 ± 0.61	0.169	1.36 ± 0.68	0.204
**Mean Lesions Attempted**	1.54 ± 0.94	1.49 ± 0.82	0.156	1.52 ± 0.83	0.465
**Mean Stents**	1.74 ± 1.23	1.73 ± 1.31	0.809	1.76 ± 1.10	0.549
**Multivessel Disease**	1275 (29.7)	205(26)	<0.001	208(28)	0.536
**Intracoronary Imaging**					
*Intravascular Ultrasound*	382 (8.9)	40 (5)	<0.001	39 (5)	0.973
*Optical Coherence Tomography*	34 (0.8)	9 (1)	0.302	2 (0.3)	0.078
**Coronary Physiology**	434 (10.1)	25 (3)	<0.001	71 (10)	<0.001
**Thrombectomy Used**	74 (1.7)	102 (13)	<0.001	61 (8)	0.006
**Glycoprotein IIb/IIIa Antagonist**	803 (18.7)	276 (36)	<0.001	206 (28)	<0.001
**Calcium Modification**					
*Rotational Atherectomy*	119 (2.8)	6 (1)	0.005	13 (2)	0.092
*Cutting Balloon*	167 (3.9)	20 (3)	0.074	26 (4)	0.314
*Intravascular Lithothripsy*	6 (0.1)	1 (0.1)	0.940	5 (0.7)	0.325
**Procedural Complications**					
*Coronary Dissection*	54 (1.3)	8 (1)	0.599	11 (1.5)	0.443
*Side Branch Occlusion*	27 (0.6)	4 (0.5)	0.711	6 (0.8)	0.491
*Perforation*	18 (0.4)	8 (1)	0.054	4 (0.5)	0.271
**IRA Flow Post**					
*TIMI 0*	20 (0.5)	58 (8)	<0.001	6 (0.8)	<0.001
*TIMI 3*	3597 (83.8)	570 (74)	<0.001	647 (86)	<0.001

TIMI = Thrombolysis in Myocardial Infarction; NS = Not Significant.

**Table 3:** In-hospital and longer-term outcomes in NSTEMI patients with unmatched TIMI 2-3 flow compared to TIMI 0-1 flow and TIMI 0-1 compared to TIMI 2-3 flow after propensity matching.

	TIMI 2-3 N = 4294	TIMI 0-1 N = 775	*p* Value	TIMI 2-3 N = 750	*p* Value
**In-Hospital Outcomes**					
Cerebrovascular Accident	3 (0.1)	0 (0)	0.463	0 (0)	1.0
Re-Infarction	5 (0.1)	1 (0.1)	0.922	1 (0.1)	0.985
Tamponade	4 (0.1)	1 (0.1)	0.766	1 (0.1)	0.985
Death	38 (0.9)	9 (1.2)	0.452	9 (1.2)	0.955
Arterial Complication	20 (0.5)	4 (0.5)	0.851	6 (0.8)	0.144
**Longer-Term Outcomes**					
1-month mortality	77 (1.8)	14 (1.8)	0.576	15 (2)	0.787
6-month mortality	151 (3.5)	28 (3.6)	0.659	34 (4.5)	0.367
12-month mortality	236 (5.5)	39 (5)	0.632	52 (6.9)	0.121
5-year mortality	845 (19.7)	124 (16)	0.019	136 (18)	0.341

Values are n (%). TIMI = Thrombolysis in Myocardial Infarction; NS = Not Significant.

**Table 4:** Cox regression analysis of 5-year outcomes after NSTEMI associated with mortality.

	Hazard Ratio	95% Confidence Interval
Age	1.07	1.05 to 1.09
Male Gender	0.98	0.68 to 1.4
Cerebrovascular Accident	1.21	0.73 to 2.03
Peripheral Arterial Disease	1.96	1.22 to 3.13
Smoking	1.07	0.75 to 1.54
Previous MI	1.25	0.82 to 1.90
Diabetes	1.99	1.35 to 2.94
Chronic Kidney Disease	2.64	1.43 to 4.87
Severe LVSD	1.99	1.07 to 3.69
Cardiogenic Shock	2.31	1.05 to 5.11
TIMI 0 Flow	1.08	0.49 to 2.32
Thrombectomy Device Usage	0.93	0.71 to 1.21
Glycoprotein IIb/IIIa	0.64	0.46 to 0.87
Left Main Stem Culprit	1.48	1.31 to 1.67
Left Anterior Descending Artery Culprit	1.15	1.05 to 1.25
Right Coronary Artery Culprit	1.12	1.04 to 1.21
Circumflex Artery Culprit	0.99	0.91 to 1.07

Values are Hazard Ration and 95% Confidence Interval. MI = myocardial infarction; TIMI = Thrombolysis in Myocardial Infarction; LVSD = left ventricular systolic dysfunction.

## Data Availability

The original contributions presented in this study are included in the article. Further inquiries can be directed to the corresponding author.

## References

[1] Licordari R., Costa F., Garcia-Ruiz V., Mamas M.A., Marquis-Gravel G., Hernandez J.M.d.l.T., Doblas J.J.G., Jimenez-Navarro M., Rodriguez-Capitan J., Urbano-Carrillo C. (2024). The Evolving Field of Acute Coronary Syndrome Management: A Critical Appraisal of the 2023 European Society of Cardiology Guidelines for the Management of Acute Coronary Syndrome. J. Clin. Med..

[2] Bode C., Zirlik A. (2007). STEMI and NSTEMI: The dangerous brothersThe opinions expressed in this article are not necessarily those of the Editors of the European Heart Journal or of the European Society of Cardiology. Eur. Heart J..

[3] Gaze D., Gaze D. (2012). Coronary Artery Disease—Current Concepts in Epidemiology, Pathophysiology, Diagnostics and Treatment. Coronary Artery Disease—Current Concepts in Epidemiology, Pathophysiology, Diagnostics and Treatment.

[4] Libby P., Theroux P. (2005). Pathophysiology of Coronary Artery Disease. Circulation.

[5] Wang T.Y., Zhang M., Fu Y., Armstrong P.W., Newby L.K., Gibson C.M., Moliterno D.J., Van de Werf F., White H.D., Harrington R.A. (2009). Incidence, distribution, and prognostic impact of occluded culprit arteries among patients with non–ST-elevation acute coronary syndromes undergoing diagnostic angiography. Am. Heart J..

[6] Polonski L., Gasior M., Gierlotka M., Osadnik T., Kalarus Z., Trusz-Gluza M., Zembala M., Wilczek K., Lekston A., Zdrojewski T. (2011). A comparison of ST elevation versus non-ST elevation myocardial infarction outcomes in a large registry database: Are non-ST myocardial infarctions associated with worse long-term prognoses?. Int. J. Cardiol..

[7] Hung C.-S., Chen Y.-H., Huang C.-C., Lin M.-S., Yeh C.-F., Li H.-Y., Kao H.-L. (2018). Prevalence and outcome of patients with non-ST segment elevation myocardial infarction with occluded “culprit” artery—A systemic review and meta-analysis. Crit. Care.

[8] Khan A.R., Golwala H., Tripathi A., Bin Abdulhak A.A., Bavishi C., Riaz H., Mallipedi V., Pandey A., Bhatt D.L. (2017). Impact of total occlusion of culprit artery in acute non-ST elevation myocardial infarction: A systematic review and meta-analysis. Eur. Heart J..

[9] Laforgia P.L., Auguadro C., Bronzato S., Durante A. (2022). The Reduction of Mortality in Acute Myocardial Infarction: From Bed Rest to Future Directions. Int. J. Prev. Med..

[10] Turnipseed S.D., Trythall W.S., Diercks D.B., Laurin E.G., Kirk J.D., Smith D.S., Main D.N., Amsterdam E.A. (2009). Frequency of Acute Coronary Syndrome in Patients with Normal Electrocardiogram Performed during Presence or Absence of Chest Pain. Acad. Emerg. Med..

[11] Ricci F., Martini C., Scordo D.M., Rossi D., Gallina S., Fedorowski A., Sciarra L., Chahal C.A., Meyers H.P., Herman R. (2025). ECG Patterns of Occlusion Myocardial Infarction: A Narrative Review. Ann. Emerg. Med..

[12] Cui K., Lyu S., Song X., Yuan F., Xu F., Zhang M., Zhang M., Wang W., Zhang D., Tian J. (2018). Effect of Coronary Collaterals on Prognosis in Patients Undergoing Primary Percutaneous Coronary Intervention for Acute ST-Segment Elevation Myocardial Infarction: A Meta-Analysis. Angiology.

[13] Meier P., Hemingway H., Lansky A.J., Knapp G., Pitt B., Seiler C. (2012). The impact of the coronary collateral circulation on mortality: A meta-analysis. Eur. Heart J..

[14] Shen Y., Wu F., Pan C., Zhu T., Zhang Q., Zhang R., Ding F., Lu L., Hu J., Yang Z. (2014). Clinical relevance of angiographic coronary collaterals during primary coronary intervention for acute ST-elevation myocardial infarction. Chin. Med. J..

[15] Hwang H., Park C., Cho J., Jin E., Sohn I.S., Kim D., Kim C. (2018). Clinical characteristics of occluded culprit arteries and collaterals in patients with non-ST-segment elevation myocardial infarction and impact on clinical outcomes. Exp. Ther. Med..

[16] Aslanger E.K., Yıldırımtürk Ö., Şimşek B., Bozbeyoğlu E., Şimşek M.A., Karabay C.Y., Smith S.W., Değertekin M. (2020). DIagnostic accuracy oF electrocardiogram for acute coronary OCClUsion resuLTing in myocardial infarction (DIFOCCULT Study). Int. J. Cardiol. Heart Vasc..

[17] McLaren J., de Alencar J.N., Aslanger E.K., Meyers H.P., Smith S.W. (2024). From ST-Segment Elevation MI to Occlusion MI: The New Paradigm Shift in Acute Myocardial Infarction. JACC Adv..

[18] Spadafora L., Pastena P., Cacciatore S., Betti M., Biondi-Zoccai G., D’aScenzo F., De Ferrari G.M., De Filippo O., Versaci F., Sciarretta S. (2025). One-Year Prognostic Differences and Management Strategies between ST-Elevation and Non-ST-Elevation Myocardial Infarction: Insights from the PRAISE Registry. Am. J. Cardiovasc. Drugs.

[19] Díaz-Herrera B.A., Roman-Rangel E., Castro-García C.A., Martinez D.S.-L., Gopar-Nieto R., Velez-Talavera K.G., Espinosa-Martínez M.P., March-Mifsut S., Latapi-Ruiz-Esparza X., Preciado-Gutiérrez Ó.U. (2025). Derivation of an artificial intelligence-based electrocardiographic model for the detection of acute coronary occlusive myocardial infarction. Arch. Cardiol. Mex..

[20] Zhai H., Li R., Zhao X., Chu Q. (2025). Acute occlusion of the LMCA without significant dynamic changes in the ECG: A case report. Medicine.

[21] Krishnaswamy A., Lincoff A.M., Menon V. (2009). Magnitude and consequences of missing the acute infarct-related circumflex artery. Am. Heart J..

[22] Morawska I., Niemiec R., Stec M., Wrona K., Bańka P., Swinarew A., Wybraniec M., Mizia-Stec K. (2021). Total Occlusion of the Infarct-Related Artery in Non-ST-Elevation Myocardial Infarction (NSTEMI)—How Can We Identify These Patients?. Medicina.

[23] Scarsini R., Terentes-Printzios D., De Maria G.L., Ribichini F., Banning A. (2020). Why, When and How Should Clinicians Use Physiology in Patients with Acute Coronary Syndromes?. Interv. Cardiol. Rev. Res. Resour..

[24] Feng X., Liu T. (2025). Identification and Management Strategies for Intracoronary High Thrombus Burden in Patients With STEMI: A Practical Experience and Literature Review. Rev. Cardiovasc. Med..

[25] Caixeta A., Lansky A.J., Mehran R., Brener S.J., Claessen B., Généreux P., Palmerini T., Witzenbichler B., Guagliumi G., Brodie B.R. (2013). Predictors of suboptimal TIMI flow after primary angioplasty for acute myocardial infarction: Results from the HORIZONS-AMI trial. EuroIntervention.

[26] Ayad S.W., El Zawawy T.H., Lotfy M.I., Naguib A.M., El Amrawy A.M. (2021). Incidence and impact of totally occluded culprit coronary artery in patients with non-ST segment elevation myocardial infarction acute coronary syndrome. Egypt. Heart J..

[27] Karwowski J., Gierlotka M., Gąsior M., Poloński L., Ciszewski J., Bęćkowski M., Kowalik I., Szwed H. (2017). Relationship between infarct artery location, acute total coronary occlusion, and mortality in STEMI and NSTEMI patients. Pol. Arch. Intern. Med..

[28] Bahrmann P., Rach J., Desch S., Schuler G.C., Thiele H. (2011). Incidence and distribution of occluded culprit arteries and impact of coronary collaterals on outcome in patients with non-ST-segment elevation myocardial infarction and early invasive treatment strategy. Clin. Res. Cardiol..

[29] Constantinides S.S., Gieowarsingh S., Halim M., Been M., Shiu M.F. (2003). Predictors of mortality in patients with acute coronary syndrome undergoing percutaneous coronary intervention. Heart.

[30] Entezarjou A., Mohammad M.A., Andell P., Koul S. (2018). Culprit vessel: Impact on short-term and long-term prognosis in patients with ST-elevation myocardial infarction. Open Heart.

[31] Waziri H., Jørgensen E., Kelbæk H., Fosbøl E.L., Pedersen F., Mogensen U.M., Gerds T.A., Køber L., Wachtell K. (2016). Acute myocardial infarction and lesion location in the left circumflex artery: Importance of coronary artery dominance. EuroIntervention.

[32] De Luca G., Bellandi F., Huber K., Noc M., Petronio A.S., Arntz H.-R., Maioli M., Gabriel H.M., Zorman S., DE Carlo M. (2011). Early glycoprotein IIb-IIIa inhibitors in primary angioplasty-abciximab long-term results (EGYPT-ALT) cooperation: Individual patient’s data meta-analysis. J. Thromb. Haemost..

[33] Svilaas T., Vlaar P.J., van der Horst I.C., Diercks G.F., de Smet B.J., Heuvel A.F.v.D., Anthonio R.L., Jessurun G.A., Tan E.-S., Suurmeijer A.J. (2008). Thrombus Aspiration during Primary Percutaneous Coronary Intervention. N. Engl. J. Med..

[34] Lagerqvist B., Fröbert O., Olivecrona G.K., Gudnason T., Maeng M., Alström P., Andersson J., Calais F., Carlsson J., Collste O. (2014). Outcomes 1 Year after Thrombus Aspiration for Myocardial Infarction. N. Engl. J. Med..

[35] Jolly S.S., A Cairns J., Yusuf S., Rokoss M.J., Gao P., Meeks B., Kedev S., Stankovic G., Moreno R., Gershlick A. (2016). Outcomes after thrombus aspiration for ST elevation myocardial infarction: 1-year follow-up of the prospective randomised TOTAL trial. Lancet.

[36] Hochman J.S., Lamas G.A., Buller C.E., Dzavik V., Reynolds H.R., Abramsky S.J., Forman S., Ruzyllo W., Maggioni A.P., White H. (2006). Coronary Intervention for Persistent Occlusion after Myocardial Infarction. N. Engl. J. Med..

